# Oxidative/Nitrosative Stress and Protein Damages in Aqueous Humor of Hyperglycemic Rabbits: Effects of Two Oral Antidiabetics, Pioglitazone and Repaglinide

**DOI:** 10.1155/2012/653678

**Published:** 2012-03-04

**Authors:** Anna Gumieniczek, Beata Owczarek, Bernadeta Pawlikowska

**Affiliations:** Department of Medicinal Chemistry, Medical University of Lublin, Jaczewskiego 4, 20-090 Lublin, Poland

## Abstract

The present study was undertaken to determine oxidative/nitrosative stress in aqueous humor of alloxan-induced hyperglycemic rabbits and to investigate the effects of two oral antidiabetic drugs, pioglitazone from peroxisome proliferator-activated receptor gamma (PPAR*γ*) agonists and repaglinide from nonsulfonylurea K_ATP_ channel blockers. Ascorbic acid (AA), glutathione (GSH), total antioxidant status (TAS), lipid peroxidation products (LPO), total nitrites (NO_2_), advanced oxidized protein products (AOPP), and protein carbonyl groups (PCG) were determined using respective colorimetric and ELISA methods. In our hyperglycemic animals, AA decreased by 77%, GSH by 45%, and TAS by 66% as compared to control animals. Simultaneously, LPO increased by 78%, PCG by 60%, AOPP by 84%, and NO_2_ by 70%. In pioglitazone-treated animals, AA and TAS increased above control values while GSH and PCG were normalized. In turn, LPO was reduced by 54%, AOPP by 84%, and NO_2_ by 24%, in relation to hyperglycemic rabbits. With repaglinide, AA and TAS were normalized, GSH increased by 20%, while LPO decreased by 45%. Our results show that pioglitazone and repaglinide differ significantly in their ability to ameliorate the parameters like NO_2_, PCG, and AOPP. In this area, the multimodal action of pioglitazone as PPAR*γ* agonist is probably essential.

## 1. Introduction

The eye is a unique organ since it is constantly exposed to radiation, atmospheric oxygen, environmental chemicals, and physical abrasion. In consequence, the eye provides a unique situation for generation of reactive oxygen species (ROS). In particular, oxidative stress is implicated in the etiology of many ocular diseases such as glaucoma, retinal degeneration, ocular inflammation, cataracts, and diabetic complications [1–3]. Also nitrogen reactive species (RNS) play an important role in different oxidative alterations [4, 5]. Nitric oxide (NO) is an important messenger in vascular and nervous systems or in immunological reactions including these in the eye. On the other hand, NO formed in excess by inducible NO synthase (iNOS) may cause serious ocular injuries [6]. 

Ocular tissues and fluids contain antioxidants that play a key role in protecting them against these oxidative/nitrosative damages. Aqueous and vitreous humors as well as lens contain high amounts of ascorbic acid (AA). It is generally accepted that it offers significant protection for the eye by suppressing generation of free radicals [7]. Also ocular glutathione (GSH) participates in neutralization of reactive species and maintains other antioxidants in their active forms. Unfortunately, these antioxidants are not able to eliminate free radicals completely and if oxidative stress is severe, it may cause cell damage or death [3].

Protein carbonyls groups (PCG), markers of early protein oxidation, and advanced oxidized protein products (AOPP) have been recently described as closely related to different pathological situations [8–10]. Some findings also suggest that the changes in proteins may be important in development of ocular diabetic complications [11].

Therefore, the present study was undertaken to determine several markers of oxidative/nitrosative stress in aqueous humor of alloxan-induced hyperglycemic rabbits where such experiments have not been performed previously. We wanted to explore potent relationship between some commonly studied parameters of oxidative alterations: AA, GSH, total antioxidant status (TAS), lipid peroxidation products (LPO), total nitrites (NO_2_), and two markers of oxidative protein modification, PCG and AOPP, and search for this which showed more expressive correlations.

It is known that structures through which aqueous humor leaves the anterior chamber may be a target of pharmacological manipulations. Thus, reducing ROS/RNS overproduction and its consequences may be an effective strategy for protection against severe ocular injuries [11, 12]. Therefore, the second goal of the present study was to investigate which of two oral antidiabetic drugs, a peroxisome proliferator-activated receptor gamma (PPAR*γ*) agonist pioglitazone or a nonsulfonylurea K_ATP_ channel blocker repaglinide could be more effective in ameliorating these oxidative/nitrosative changes. Pioglitazone belonging chemically to 2,4-thiazolidinediones family (TZDs) acts in diabetes mainly by decreasing insulin resistance at the level of the muscle and liver. It has been shown to be potent antioxidant in different pathological situations connected with oxidative/nitrosative stress including diabetes [13, 14]. Repaglinide, from nonsulfonylurea K_ATP_ channel blockers, is a carbamoyl methyl benzoic acid derivative with the effect on early insulin secretion and reducing postprandial glucose directly [15]. Additionally, some results from the literature have previously demonstrated that it may have positive effects on parameters of oxidative/nitrosative stress [16, 17].

## 2. Material and Methods

### 2.1. Animals and Chemicals

White male New Zealand rabbits (the mean weight 3.1 kg) were housed in a controlled environment with 12 h light and dark cycles. They were provided with standard diet and water *ad libitum*. Animal care was in accordance with the “Principles of Laboratory Animal Care” (NIH publication No. 86-23, revised 1985) and with the Guidelines of Medical University of Lublin Animal Ethics Committee. The rabbits were divided into six groups of 5 animals: normal control (Group C), control treated with pioglitazone (Group CP), control treated with repaglinide (Group CR), hyperglycemic (Group H), hyperglycemic treated with pioglitazone (Group HP), and hyperglycemic treated with repaglinide (Group HR). Hyperglycemia was induced by a single intravenous injection of 80 mg/kg of alloxan. Two weeks after the injection when hyperglycemia was verified by blood glucose concentration higher than 11 mmol/L, administration of pioglitazone at a dose of 1 mg/kg and repaglinide at a dose of 0.3 mg/kg was started and continued for 4 weeks (the start of experiment). The drugs were given directly to oral cavity using a syringe without a needle, every day before the morning feeding. During experiment, glucose concentration was monitored once a week. At the end of experiment, the animals were sacrificed with pentobarbital sodium at a dose of 60 mg/kg. The aqueous humor was collected by a 26-G needle attached to a tuberculin syringe with special care to avoid blood contamination. The needle was introduced into anterior chamber and ca. 100 *μ*L aqueous humor was withdrawn. The samples were stored at −70°C until analysis.

TAS was assessed using a commercially available test from Randox Laboratories Ltd. (UK) while AA estimation was performed according to Kyaw [18]. GSH and LPO were determined using Bioxytech GSH-400 and LPO-586 kits from Oxis Research (USA). The stable metabolites of NO were estimated using a Nitrate/Nitrite Assay Kit from Fluka Chemicals (UK). Nitrates were reduced to nitrites by incubation of each sample for 120 min in the presence of nitrate reductase and NADPH. Then, total nitrites were assayed by adding of Griess reagent and measuring the absorbance at 540 nm. PCG and AOPP levels were determined using respective ELISA or colorimetric kits from Immundiagnostik AG (Germany). Protein content was determined by the method of Lowry et al. [19] using bovine serum albumin as standard.

Two spectrophotometers, UV-Vis CE-6000 from CECIL Instruments (UK) and a Microplate Reader PowerWave XS from Bio-Tek Instruments Inc. (USA), were used.

### 2.2. Statistical Analysis

All numerical data are presented as the mean with respective standard error (SEM). The significance of differences was determined with Kruskal-Wallis and Mann-Whitney's *U* tests. Multiple regression and Spearman's rank coefficients were used to investigate the possible correlations between AOPP or PCG level and other parameters. Probability *P* less than 0.05 were considered significant. For all statistical evaluation, statistica software was used.

## 3. Results

In hyperglycemic animals, there were decreases of AA by 77%, GSH by 45%, and TAS by 66% as compared to control animals. Simultaneously, there were increases of LPO by 78%, PCG by 60%, AOPP by 84%, and NO_2_ by 70%. Pioglitazone increased AA and TAS above control values and normalized GSH and PCG. In turn, LPO was reduced by 54%, AOPP by 84% and NO_2_ by 24% in relation to hyperglycemic rabbits. With repaglinide treatment, AA and TAS were normalized while GSH increased by 20% and LPO decreased by 45% in relation to hyperglycemic rabbits. However, repaglinide did not affect the levels of PCG, AOPP, and NO_2_ (Table 1).

**Figure 1:**
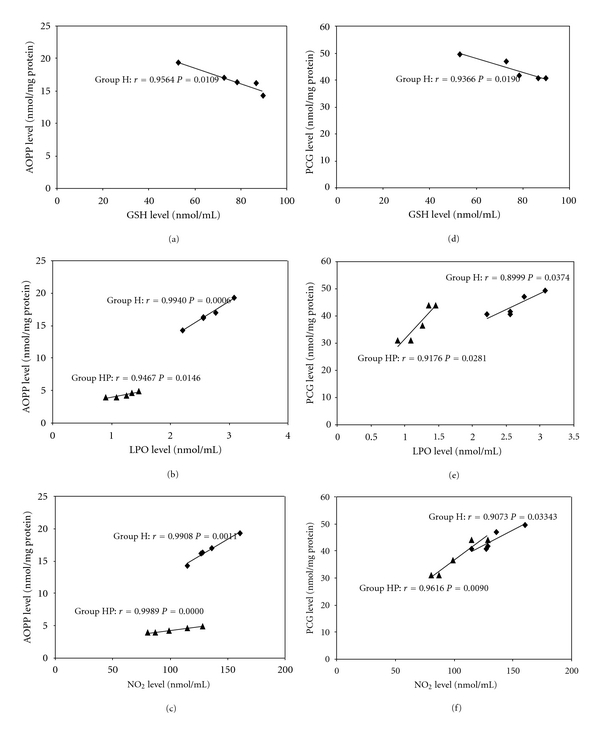
Correlations between GSH, LPO, and NO_2_ and respective protein oxidation marker, AOPP ((a)–(c)) or PCG ((d)–(f)), in hyperglycemic (Group H) and hyperglycemic pioglitazone-treated (Group HP) animals.

Multiple regression analysis did not show significant correlations. However, some correlations were observed when respective pairs of parameters were taken into account. In hyperglycemic group, we observed a negative correlation between AOPP and GSH, and positive correlations in pairs AOPP-LPO and AOPP-NO_2_ (Figures 1(a)–1(c)). In hyperglycemic pioglitazone-treated group, similar correlations were observed between AOPP and LPO, and AOPP and NO_2_. In the case of PCG, correlations for both hyperglycemic and hyperglycemic pioglitazone-treated animals were also significant, although respective r values were lower than these for AOPP (Figures 1(d) and 1(e)). Because repaglinide did not affect the altered protein oxidation, in this case respective correlations were not calculated.

## 4. Discussion

In the eye of diabetic subjects, the occurrence of oxidative stress was demonstrated as depletion of GSH, an important aqueous antioxidant [20]. Between others, GSH maintains the second major antioxidant AA in its active form [21]. Relation between these two antioxidants was confirmed in the present study where diminished levels of GSH and AA were stated in aqueous humor of our hyperglycemic animals. Some reports suggest that intracellular GSH level may be decreased by RNS derived from NO produced in excess by iNOS. As a consequence, production of nitrosoglutathione and formation of protein-mixed disulfides with glutathione have been reported [22]. Previously, excessive levels of NO were stated in vitreous fluid of patients with proliferative diabetic retinopathy [6]. It was also observed in the present study together with a decreased level of GSH. The altered antioxidant balance in aqueous humor of our hyperglycemic rabbits was additionally confirmed by a significant increase in lipid peroxidation. Previously, elevated concentrations of LPO were stated in vitreous fluid of diabetic db/db mice [23] and in aqueous fluid of glaucoma patients [20].

Under situation of oxidative stress, many attention is devoted to oxidative damages in proteins [24]. They may result, between others, from formation of different protein adducts. Early modifications often bring carbonyl groups to proteins and may be estimated as 2,4-dinitrophenylhydrazine derivatives or PCG [25]. Recently, a large interest lies on latter products of protein oxidation (AOPP) which are defined as dityrosine-containing cross-linked protein products [10, 26]. In aqueous humor of our hyperglycemic animals, the both markers, PCG and AOPP, were elevated in comparison with respective controls. Previously, similar changes were found in plasma of type 2 diabetic patients where higher increase in the level of AOPP compared with PCG was found. It was explained by lower susceptibility of AOPP cross-linked proteins to proteolysis and their subsequent accumulation in plasma [10]. In the present study, we additionally concluded that AOPP correlated better than PCG to other examined parameters (Figure 1).

In our study AA, TAS, GSH, and LPO were ameliorated by the two drugs, pioglitazone and repaglinide. However, only after pioglitazone, significant changes in NO_2_, PCG, and AOPP were observed. It is known that AOPP are formed by action of chloraminated oxidants, mainly hypochlorous acid and chloramines, produced by myeloperoxidase (MPO) [26, 27]. On the other hand, it has been showed that pioglitazone inhibits proinflammatory factors including MPO. Therefore, its present effect on AOPP in aqueous humor of hyperglycemic animals may be, at least in part, a consequence of the above ability.

It is known that expression of many genes including proinflammatory cytokines, adhesion molecules, and others such as iNOS was regulated by a nuclear factor NF-*κ*B. It is also supposed that beneficial effects of antioxidants against oxidative complications may involve effective inhibition of NF-*κ*B [6]. Such mechanism for antioxidative and anti-inflammatory properties of PPAR*γ* agonists, including pioglitazone, has been proposed [28–30]. Previous data about decreased levels of NO_2_ and MPO after pioglitazone in lung and testis of our hyperglycemic animals confirm this [31, 32]. However, the exact mechanism whereby the drug exerts its antioxidant effect is still unclear. In this area, the multimodal action of PPAR*γ* agonists is probably essential [33].

As far as concerning K_ATP_ channel blockers, it cannot be excluded that they may affect oxidative/nitrosative stress. After repaglinide, total serum antioxidant capacity [17] and lipid peroxidation were ameliorated [15, 16]. After another drug mitiglinide, significant decreases of lipid peroxidation and nitrotyrosine were also observed [34]. However, all these effects occurred in type 2 diabetes where they could be probable by better controlling postprandial hyperglycemia and thus by the cluster of oxidative stress. Additionally, it has been stated that insulin *per se* reduces the level of NF-*κ*B. Because NF-*κ*B regulates the expression of enzymes involved in ROS/RNS generation, in this way, insulin can modulate the mechanisms involved in oxidative/nitrosative stress [35]. In our previous studies concerning heart, lung, and testis, repaglinide significantly affected nitrotyrosine and LPO levels but did not affect NO_2_ and PCG levels [31, 32]. It was confirmed in the present study by the lack of any effect on PCG, AOPP, and NO_2_.

What is important in the present study is that pioglitazone and repaglinide did not significantly affect glucose concentration in our hyperglycemic animals. After alloxan injection, these animals preserved insulin secretion but its amount was very low because of destruction of many B cells by alloxan. Therefore, repaglinide could not stimulate its secretion in sufficient way and pioglitazone failed to affect its sensitivity (Table 2). This lack of antihyperglycemic activity was expected and used by us to differentiate, at least in part, some direct antioxidative/antinitrosative effects of the drugs from effects mediated via increased insulin action. When animals with type 2 diabetes were used, these two kinds of effects could not be separated.

## 5. Conclusions

In aqueous humor of our hyperglycemic animals, the decreased AA, GSH, and TAS as well as increased LPO, NO_2_, PCG, and AOPP were observed confirming the exposition of ocular structures on oxidative/nitrosative stress. It was observed that correlations between AOPP and other factors were more expressive than the same correlations for PCG. We concluded that AOPP which was easy to obtain, corresponded better than PCG to other examined parameters. In addition, antioxidative/antinitrosative properties of pioglitazone and repaglinide were compared. The results obtained in the present study confirm that pioglitazone and repaglinide differ significantly in their ability to ameliorate the examined markers of oxidative/nitrosative stress in aqueous humor, especially in respect to protein modifications. It cannot be excluded that K_ATP_ channel blockers like repaglinide may affect oxidative/nitrosative stress but PPAR*γ* agonists like pioglitazone seem to act more comprehensively which was confirmed independently on their action oh hyperglycemia.

## Figures and Tables

**Table 1:** Effects of pioglitazone and repaglinide on oxidative/nitrosative stress parameters in the aqueous humor of control and hyperglycemic rabbits.

	Group C	Group CP	Group CR	Group H	Group HP	Group HR
TAS (mmol/mL)	1.99 ± 0.10	1.96 ± 0.15	1.90 ± 0.15	0.67 ± 0.09^a^	2.37 ± 0.14^a,b^	1.56 ± 0.15^b^
AA (*μ*g/mL)	12.90 ± 1.05	13.94 ± 0.93	10.03 ± 1.05	3.02 ± 0.38^a^	21.87 ± 1.18^a,b^	12.62 ± 1.36^b^
GSH (nmol/mL)	138.7 ± 4.49	144.2 ± 12.84	117.7 ± 3.32^a^	76.14 ± 6.56^a^	131.4 ± 5.86^b^	91.06 ± 8.17^a,b^
NO_2_ (nmol/mL)	39.04 ± 2.84	43.67 ± 4.09	37.44 ± 2.5	113.4 ± 7.63^a^	102.0 ± 8.78^a^	142.5 ± 2.54^a^
LPO (nmol/mL)	0.57 ± 0.27	0.89 ± 0.25	0.68 ± 0.023	2.64 ± 0.14^a^	1.21 ± 0.11^a,b^	1.45 ± 0.17^a,b^
PCG (nmol/mg protein)	17.86 ± 1.36	16.98 ± 1.30	24.13 ± 1.63^a^	44.49 ± 2.15^a^	20.46 ± 2.96^b^	40.46 ± 3.57^a^
AOPP (nmol/mg protein)	2.53 ± 0.26	2.44 ± 0.26	2.76 ± 0.32	16.15 ± 2.24^a^	4.31 ± 0.18^a,b^	19.29 ± 0.88^a,b^

Values are mean ± SEM (*n* = 5). TAS: total antioxidant status, AA: ascorbic acid, GSH: glutathione, NO_2_: total nitrites, PCG: protein carbonyls, AOPP: advanced oxidized protein products. C: control rabbits, CP: control rabbits treated with pioglitazone, CR: control rabbits treated with repaglinide, H: hyperglycemic rabbits, HP: hyperglycemic rabbits treated with pioglitazone, HR: hyperglycemic rabbits treated with repaglinide. ^a^Significant at *P* < 0.05 versus Group C. ^b^Significant at *P* < 0.05 versus Group H.

**Table 2:** Blood glucose and plasma insulin concentrations in control and hyperglycemic rabbits at the start and the end of experiment.

Group	Glucose (mmol/L)		Insulin (mU/L)	
	Start	End	Start	End
Control (C)	6.2 ± 0.1	5.7 ± 0.3	13.16 ± 1.26	13.30 ± 1.12
Control-pioglitazone (CP)	6.5 ± 0.3	5.9 ± 0.3^b^	11.74 ± 0.72	14.12 ± 0.98^b^
Control-repaglinide (CR)	6.3 ± 0.2	4.0 ± 0.3^a,b,∗^	12.83 ± 1.01	20.0 ± 1.42^a,b,∗^
Hyperglycemic (H)	26.3 ± 2.3^a^	24.9 ± 2.8^a^	3.21 ± 0.63^a^	2.79 ± 0.79^a^
Hyperglycemic-pioglitazone (HP)	27.2 ± 0.3^a^	23.9 ± 1.8^a^	2.31 ± 0.15	2.01 ± 0.34^a^
Hyperglycemic-repaglinide (HR)	26.4 ± 1.2^a^	24.0 ± 2.3^a^	2.32 ± 0.15^a^	2.02 ± 0.04^a^

Values are mean ± SEM (*n* = 5). ^a^Significant at *P* < 0.05 versus Group C. ^b^Significant at *P* < 0.05 versus Group H. *Significant at *P* < 0.05 versus start.
